# Supplementary material for: Multi‐dataset identification of innovative feature genes and molecular mechanisms in keratoconus
Source: J Cell Mol Med. 2024 Sep 19;28(18):e70079. doi: 10.1111/jcmm.70079 (PMC11412914; doi:10.1111/jcmm.70079)
Supplement: Supplementary file 2 — Material S2. [file JCMM-28-e70079-s002.zip › 7.GSEA analysis/1.ARL11/KEGG_ALPHA_LINOLENIC_ACID_METABOLISM.html]

Details for gene set KEGG\_ALPHA\_LINOLENIC\_ACID\_METABOLISM[GSEA]

|  || Dataset | ARL11.group.cls#h\_versus\_l |
| Phenotype | group.cls#h\_versus\_l |
| Upregulated in class | l |
| GeneSet | KEGG\_ALPHA\_LINOLENIC\_ACID\_METABOLISM |
| Enrichment Score (ES) | -0.4541397 |
| Normalized Enrichment Score (NES) | -1.0714315 |
| Nominal p-value | 0.37148595 |
| FDR q-value | 1.0 |
| FWER p-Value | 0.998 |
Table: GSEA Results Summary

  

Fig 1: Enrichment plot: KEGG\_ALPHA\_LINOLENIC\_ACID\_METABOLISM      
 Profile of the Running ES Score & Positions of GeneSet Members on the Rank Ordered List

  

| SYMBOL | TITLE | RANK IN GENE LIST | RANK METRIC SCORE | RUNNING ES | CORE ENRICHMENT || 1 | PLA2G4E | na | 4751 | 0.174 | -0.0473 | No |
| 2 | PLA2G3 | na | 6310 | 0.129 | -0.0257 | No |
| 3 | FADS2 | na | 7752 | 0.097 | -0.0171 | No |
| 4 | PLA2G12B | na | 9215 | 0.072 | -0.0223 | No |
| 5 | ACOX1 | na | 9236 | 0.071 | 0.0139 | No |
| 6 | PLA2G2C | na | 13424 | 0.024 | -0.0946 | No |
| 7 | JMJD7-PLA2G4B | na | 14509 | 0.015 | -0.1181 | No |
| 8 | PLA2G4A | na | 17245 | -0.005 | -0.1943 | No |
| 9 | PLA2G6 | na | 17692 | -0.008 | -0.2030 | No |
| 10 | PLA2G12A | na | 17822 | -0.009 | -0.2020 | No |
| 11 | ACOX3 | na | 18292 | -0.012 | -0.2092 | No |
| 12 | PLA2G2E | na | 19951 | -0.023 | -0.2449 | No |
| 13 | PLA2G10 | na | 23788 | -0.060 | -0.3246 | No |
| 14 | PLA2G2D | na | 27985 | -0.130 | -0.3782 | Yes |
| 15 | PLA2G2F | na | 30623 | -0.208 | -0.3467 | Yes |
| 16 | PLA2G5 | na | 32093 | -0.264 | -0.2527 | Yes |
| 17 | PLA2G2A | na | 32310 | -0.275 | -0.1171 | Yes |
| 18 | PLA2G1B | na | 33526 | -0.362 | 0.0346 | Yes |
Table: GSEA details [plain text format]

  

Fig 2: KEGG\_ALPHA\_LINOLENIC\_ACID\_METABOLISM      
 Blue-Pink O' Gram in the Space of the Analyzed GeneSet

  

Fig 3: KEGG\_ALPHA\_LINOLENIC\_ACID\_METABOLISM: Random ES distribution      
 Gene set null distribution of ES for **KEGG\_ALPHA\_LINOLENIC\_ACID\_METABOLISM**
